# Dissecting the microvascular contributions to diffuse correlation spectroscopy measurements of cerebral hemodynamics using optical coherence tomography angiography

**DOI:** 10.1117/1.NPh.8.2.025006

**Published:** 2021-04-25

**Authors:** James H. Jang, Krystyna Solarana, Daniel X. Hammer, Jonathan A. N. Fisher

**Affiliations:** a Center for Devices and Radiological Health, U. S. Food and Drug Administration, Silver Spring, Maryland, United States; b New York Medical College, Department of Physiology, Valhalla, New York, United States

**Keywords:** cerebral blood flow, diffuse correlation spectroscopy, optical coherence tomography angiography, multimodal validation, microvasculature

## Abstract

**Significance:** Diffuse correlation spectroscopy (DCS) is an emerging noninvasive, diffuse optical modality that purportedly enables direct measurements of microvasculature blood flow. Functional optical coherence tomography angiography (OCT-A) can resolve blood flow in vessels as fine as capillaries and thus has the capability to validate key attributes of the DCS signal.

**Aim:** To characterize activity in cortical vasculature within the spatial volume that is probed by DCS and to identify populations of blood vessels that are most representative of the DCS signals.

**Approach:** We performed simultaneous measurements of somatosensory-evoked cerebral blood flow in mice *in vivo* using both DCS and OCT-A.

**Results:** We resolved sensory-evoked blood flow in the somatosensory cortex with both modalities. Vessels with diameters smaller than 10  μm featured higher peak flow rates during the initial poststimulus positive increase in flow, whereas larger vessels exhibited considerably larger magnitude of the subsequent undershoot. The simultaneously recorded DCS waveforms correlated most highly with flow in the smallest vessels, yet featured a more prominent undershoot.

**Conclusions:** Our direct, multiscale, multimodal cross-validation measurements of functional blood flow support the assertion that the DCS signal preferentially represents flow in microvasculature. The significantly greater undershoot in DCS, however, suggests a more spatially complex relationship to flow in cortical vasculature during functional activation.

## 1 Introduction

Neurons are among the most metabolically active cells in the body. Although the brain accounts for just 2% of the total body weight, it consumes roughly 20% of the available oxygen in the body.^1^ Given this significant metabolic burden, preserving normal cerebral hemodynamics is critical for brain health. Pathological perturbation of any portion of the vasculature network fundamentally alters the ability to supply neurons with energy on demand,^2^ and blockage of even single microvessels can result in larger-scale changes in flow.^3^ Noninvasive clinical methods for monitoring cerebral blood flow are therefore critical for patients who have conditions, such as acute ischemic stroke,^4^^–^^6^ neurodegenerative disease,^7^ or brain injury,^8^^–^^11^ among many others.

Current clinical methods for directly monitoring cerebral blood flow include positron emission tomography utilizing H2O15 (or $C^{15}O_{2}$),^12^ MRI utilizing arterial spin labeling^13^ or dynamic contrast bolus tracking,^14^ and dynamic or xenon-enhanced computed tomography (CT).^15^ While these methods can provide quantitative or even absolute portraits of blood flow, they are not practical for continuous bedside monitoring. In addition, implementation of these modalities is limited by cost, and small hospitals, particularly in developing nations, do not have such infrastructure. Furthermore, these existing technologies inherently detect cerebral blood flow changes in relatively large vessels, rather than in microvasculature.

Diffuse correlation spectroscopy (DCS) is an emerging near-infrared optical technology that can directly measure blood flow.^16^^,^^17^ The technology is particularly promising for neurological applications because it is supposedly sensitive to flow in small blood vessels due to the high absorption (and thus low probability of photon escape) in larger blood vessels. Whereas functional near-infrared spectroscopy (fNIRS) reports brain activation based on the relative concentrations of oxygenated and deoxygenated hemoglobin, DCS monitors flow dynamics directly by observing rapid temporal intensity fluctuations in light that is scattered by tissue. DCS can therefore noninvasively resolve cerebral blood flow changes in response to even mild somatosensory stimuli.^18^

DCS has been validated in both human and *in vivo* animal experiments using a variety of established clinical techniques for monitoring cerebral blood flow such as transcranial Doppler ultrasound,^19^^–^^21^ phase-encoded velocity mapping MRI,^22^ arterial-spin labeled MRI,^23^ Xenon-CT,^9^ indocyanine green tracers,^24^ and fluorescent microsphere flow tracers.^10^ However, most of these techniques do not operate with the micron-level resolution necessary to visualize fine capillaries and those that do are invasive. Furthermore, these validation methods cannot directly discern flow in vessels as a function of diameter. This is particularly important because vessels of different diameters and at different cortical depths respond to neural activity with varied amplitudes and waveforms.^25^^,^^26^ The fidelity with which DCS signals align with a microscale portrait of blood flow in the layers of the cerebral cortex therefore remains to be directly quantified.

Optical coherence tomography (OCT) is a cross-sectional and volumetric interferometric imaging modality that has expanding applications in neurology, principally via the retina.^27^^–^^29^ OCT-angiography (OCT-A) is an extension of optical coherence tomography, an interferometric technique that allows direct access to phase changes in scattered light, thus offering a direct measure of volumetric flow velocity changes.^30^ OCT-A is particularly useful for studying neurovascular coupling because, in addition to cellular-level structural resolution, it is able to efficiently separate vascular flow from parenchymal optical tissue scatter, producing high contrast visualization of all vessels within a probed volume.^31^^–^^35^

To parse the various potential contributions to the DCS signal, we established an optical approach that permitted us to perform both macro- and microresolution optical monitoring *in vivo*. We performed DCS measurements of somatosensory-evoked changes in cerebral blood flow together with simultaneous, high-resolution functional imaging of blood flow changes using OCT-A. By using sensory stimuli, we aimed to preserve the diversity of flow patterns among the heterogeneous cortical vasculature. Ultimately, wider clinical adoption of DCS depends on a clearer understanding of the neurovascular underpinnings of the signal. Combining DCS and OCT-A offers a unique opportunity to directly visualize this precise relationship.

## 2 Methods and Materials

### 2.1 Surgical Procedures

All animal experiments were performed with a study protocol approved by and conducted in accordance with the guidelines of the White Oak Institutional Animal Care and Use Committee. Optical measurements were performed on 13 male C57BL/6J mice (12- to 24-weeks old). Anesthesia was induced by an initial exposure to 4% isoflurane (vaporized in medical grade compressed oxygen) for <30  s. Animals were additionally administered an injection of xylazine (18  mg/kg, IP) to provide a stable plane of anesthesia at low isoflurane concentrations (0.1% to 0.2%) for the remainder of the experimental session, which typically lasted 3 to 6 h. Maintenance doses of xylazine (6  mg/kg) were administered once every ∼2.5  h. This anesthesia regimen mitigated the vasodilation effects known to occur with isoflurane administration. Following initial anesthesia induction, animals were positioned in a stereotaxic apparatus (David Kopf Instruments, California). Their body temperature was measured and maintained at 37°C with a closed-loop temperature-controlled heating pad (Model TC-1000, CWE). Respiratory rate was also monitored and maintained at ∼100  breaths/min during the surgical and experimental procedures. Skin incisions were infused with lidocaine, and the eyes were covered with ointment (Lacri-Lube) to prevent drying. A midline sagittal incision was made in the skin, which exposed the coronal and lambdoid sutures on the skull. The intersection of these sutures with the midline (i.e., bregma and lambda) served as a landmark for recording locations and was also used as a guide for surgery.

**Fig. 1:**
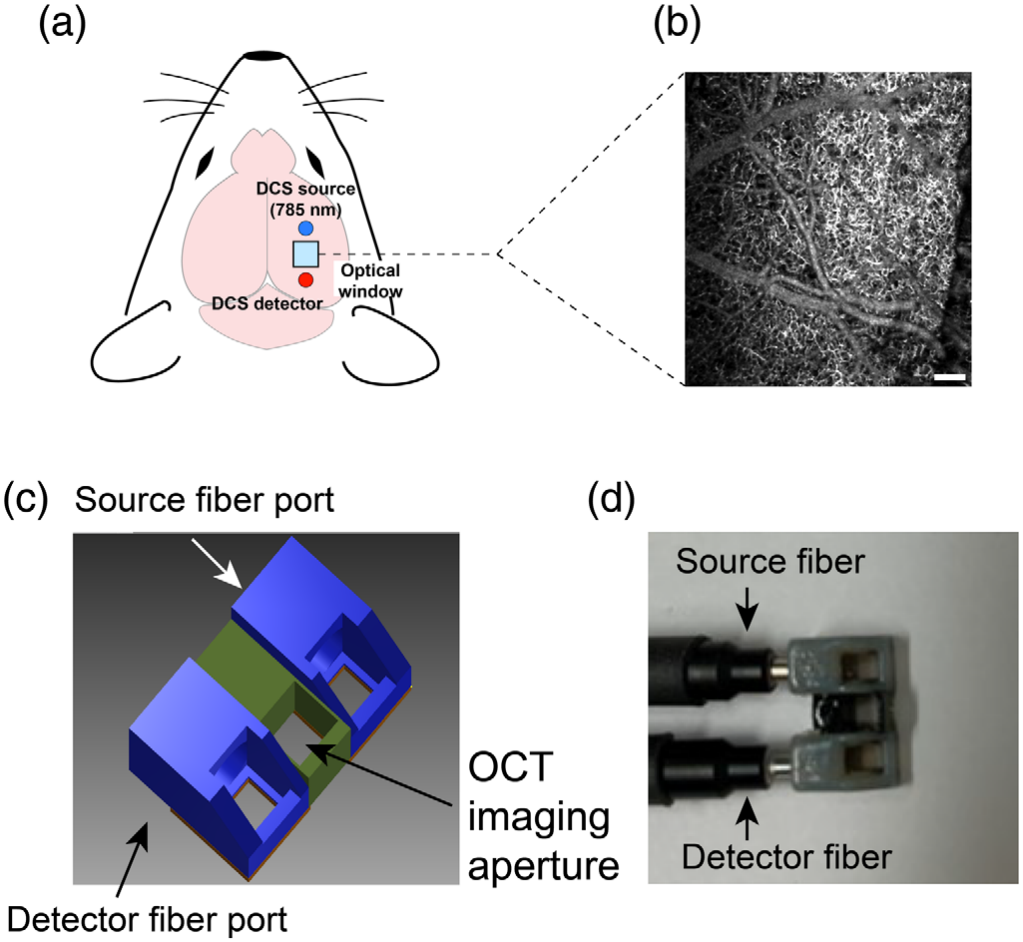
Overview of imaging region and approach for identifying regions of sensory activation. (a) The location of the recording window (blue square) on the head of a mouse relative to the underlying brain regions. The blue and red dots adjacent to the window correspond to the emitter and detector fibers from the DCS apparatus. (b) A representative *en face* OCT-A image of the cortical vasculature. Scalebar=200  μm. (c) The custom 3D-printed probe for simultaneous DCS and OCA imaging. (d) A photograph of a completed probe assembly.

A craniotomy (∼2.5×3  mm) was performed using a handheld dental drill (Osaka) and a 2×2.5  mm window (sterilized #0 cover glass) was placed over the somatosensory cortex [Fig. 1(a)]. The gap between the outer edge of the glass window and the inner edge of the craniotomy was filled with surgical silicone adhesive (Kwik-Sil, World Precision Instruments, Florida), and the coverslip was then sealed to the skull with instant adhesive gel (Loctite 454, HenKel, Wisconsin). A metal head-bar was attached to the skull contralateral to the craniotomy by dental cement (C&B Metabond, Parkell, New York). Animals recovered overnight. The transparent region of the cortex beneath the window was ∼2×2  mm.

### 2.2 Evoking Sensory Responses

The median nerve contralateral to the recording location was stimulated via a pair of 27-gauge stainless-steel needles inserted subcutaneously into the forelimb of the animal. The electrical stimulus consisted of a train of 12 current pulses generated by a constant current stimulator (DS7A, Digitimer) with the following characteristics: amplitude 1-4 mA, frequency 3 Hz, pulse duration 200  μs, and total pulse-train duration 4 s. Individual measurements of sensory-evoked changes in cerebral blood flow were separated by 30 s, which we empirically found to be the minimum intertrial duration that did not elicit monotonic alterations in the steady-state flow. At this temporal separation between trials, we did not observe habituation effects over the full duration of any preparation. To further avoid any potential habituation effects, the stimulus current was randomly selected between consecutive trials.

### 2.3 DCS System

The basic theory underlying DCS has been extensively described in previous publications.^16^^,^^31^ Briefly, when laser light migrates through tissue, the emerging intensity pattern, called a speckle pattern, is composed of bright and dark spots, which are caused by constructive and destructive interference of photons that traverse different path lengths. The intensity fluctuations of a single region, which are caused by interactions of scattered light with moving particles (i.e., red blood cells), can be used to extract information about blood flow. In particular, it is the autocorrelation decay that correlates with blood flow. Our DCS system has been previously described.^36^ Briefly, it consisted of a long coherence length continuous-wave near-infrared laser (785 nm, CrystaLaser, Nevada), a photon-counting avalanche photodiode (APD) (SPCM-AQRH-12-FC, Excelitas, Quebec, Canada), and an autocorrelator signal processing board (Correlator.com, New Jersey). NIR excitation light was delivered to the brain with a multimode optical fiber (200-μm core diameter, Thorlabs, New Jersey), and the scattered light was detected with a single-mode fiber (5-μm core diameter) connected to the APD. The sampling rate of the system was 4.4 Hz.

### 2.4 Imaging Probe Design for Simultaneous DCS and OCT-A

To permit simultaneous DCS and OCT-A angiographic portraits of blood flow, we fabricated a custom probe from semiflexible acrylate polymer using a three-dimensional printer (Objet 260 Connex3 printer, Stratasys, Minnesota) [Figs. 1(c) and 1(d)]. For the DCS modality, embedded microprisms (2 mm, Edmund Optics Inc., New Jersey) directed illumination light from the source fiber down to the head and backscattered light to the detector fiber. Midway between the source and detector fiber ports, which were separated by 5-mm, a 2  mm×2  mm aperture provided access for the OCT-A beam. All OCT-A images collected during stimulation were acquired through the aperture. During experiments, the probe was positioned such that the central clearing was directly above the implanted cranial window and sealed with silicone (Kwik-Cast, Silicone Elastomer, World Precision Instruments, Florida).

The design of the probe was guided by several factors. At the most basic level, we needed to ensure that the centroid of the forelimb representation on the cortex could be fully observed within the imaging window.^37^ This region of activation has a diameter of ∼2  mm; given preparation-to-preparation variability, we wanted to ensure that errors in window placement would not cause the probe to entirely occlude the response centroid. Another constraint, however, was that we wanted the OCT-A scan depth to match to the depth of DCS maximum sensitivity, which is influenced by the source–detector separation. Additional constraints on the probe geometry included the dimensions of the fibers themselves as well as the fact that the probe assembly needed to accommodate the window for OCT imaging. As a compromise, a source–detector separation of 5 mm was selected, which, based on previous studies, likely keeps relative blood flow measurement error below 12%.^38^

### 2.5 Modeling the DCS and OCT-A Sensory Evoked Response Waveforms

To extract quantitative features from the sensory evoked optical signals, we fit the observed waveforms to a canonical hemodynamic response function that uses two gamma density functions to approximate the hemodynamic response,^39^ i.e.,

$$\mathrm{HRF}(t)=A(\frac{t^{\alpha_{1}-1}\beta_{1}^{\alpha_{1}}e^{-\beta_{1}t}}{\Gamma (\alpha_{1})}-c\frac{t^{\alpha_{2}-1}\beta_{2}^{\alpha_{2}}e^{-\beta_{2}t}}{\Gamma (\alpha_{2})}),$$

where Γ represents the gamma function, and A, c, $\alpha_{1}$, $\alpha_{2}$, $\beta_{1}$, and $\beta_{2}$ are fitting coefficients, which were fit using the Levenberg–Marquardt algorithm in Matlab. A criterion of $R^{2}>0.9$ was used in the fitting process. Here, A controls the amplitude, c determines the ratio of the response to undershoot, and $\alpha_{1}$, $\alpha_{2}$, $\beta_{1}$, and $\beta_{2}$ control the shape of the fit function. We used the fits to quantify the response maximum and minimum as well as to define the temporal span of positive peaks and subsequent undershoots. In particular, the maximum of the positive peak and minimum of the undershoot from a waveform fit were used to calculate the response magnitudes. The temporal delineations of positive and negative peaks were used to calculate the integrated blood flow results that are summarized in Fig. 7.

### 2.6 OCT-A System and Image Processing

**Fig. 2:**
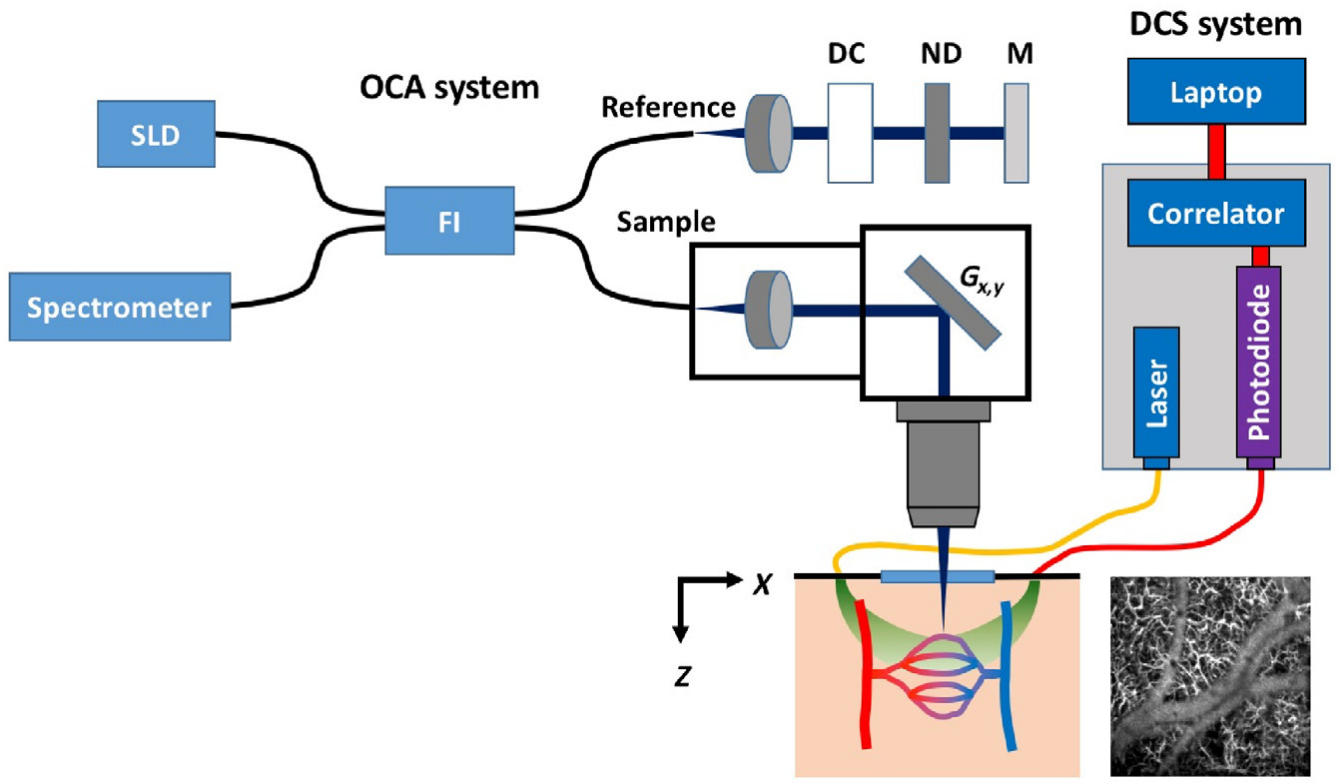
Hybrid optical apparatus (simplified) for simultaneous DCS and OCT-A. DC, dispersion compensation cube; FI, fiber interferometer; $G_{x,y}$, galvanometer scanners; M, mirror; ND, neutral density filter; SLD, superluminescent diode. The two modalities are combined through a custom 3D-printed probe placed on the region of the skull above the primary somatosensory cortex at a location coinciding with the forepaw representation. The sample is here represented by a depiction of a capillary network.

The OCT-A device (Fig. 2) used herein has been described previously,^34^^,^^40^ apart from several key modifications described below for synchronization to the DCS instrument. The system uses a 1315 nm (center wavelength) superluminescent diode light source (Exalos, Schlieren, Switzerland) with a 3-dB bandwidth of 85 nm, which produces a theoretical axial resolution in air of 9  μm. A custom-designed spectrometer (Wasatch Photonics Inc., Durham, North Carolina) with a 76-Hz line rate InGaAs linear detector (Sensors Unlimited Inc., Princeton, New Jersey) achieves a depth roll-off of 1.8  dB/mm (in the range 0 to 2.5 mm). In configuring the system optics, we had to balance the lateral spot size required to resolve capillaries with a moderate depth-of-focus to match the volume from which the majority of the DCS signal derives. Therefore, we used a 19-mm focal length source collimator to produce a ∼3-mm input beam diameter and a 10× telecentric objective (LSM02, 7.5 mm working distance, Thorlabs Inc., Newton, New Jersey). The lateral resolution measured with a nanoparticle-embedded point spread function phantom was ∼9  μm (FWHM) and the depth-of-focus was ∼500  μm.

Custom OCT system software written in LabVIEW (National Instruments, Austin, Texas) was developed to control hardware, including source power control, galvanometers for scanning, data acquisition boards for waveform generation, and cameras and frame grabbers for image acquisition. The acquired OCT images were processed in near real-time on the system computer using custom CUDA-based code running on the graphics processing unit (GPU) of a high-performance video card (GeForce GTX-760, NVIDIA). For this study, a real-time axial motion correction algorithm was implemented in the GPU software, based upon subpixel alignment of adjacent A-scans using cross-correlation.

**Fig. 3:**
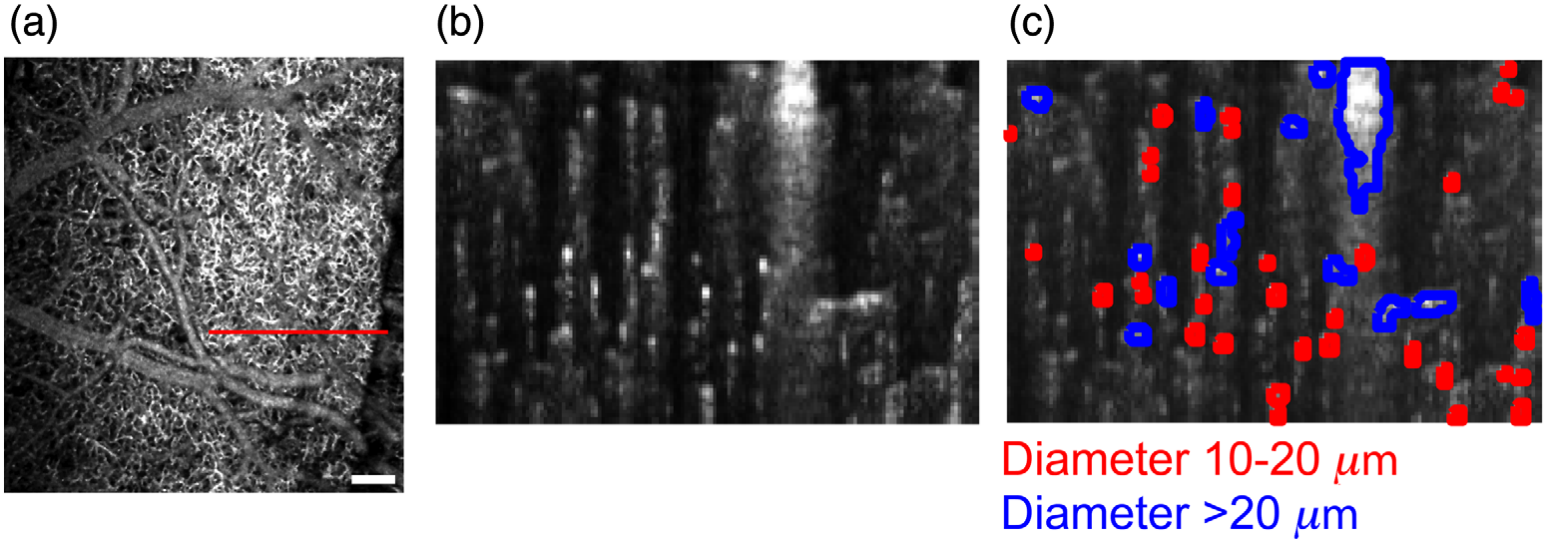
Method for dissecting OCT-A signal contributions with Otsu thresholding. (a) A sample *en face* OCT-A image. Scalebar=200  μm. The red line indicates the location selected for collection of high-resolution B-scans. (b) The B-scan corresponding to the red line drawn in (a). The bright punctae correspond to cross-sections of small blood vessels. Superimposed on this B-scan image, (c) two populations of vessel cross-sections selected from among the regions identified using Otsu’s method. In particular, vessels of diameter 10 to 20  μm (in red) and >20  μm (in blue) are shown.

Two OCT videos were created for each acquisition: a reflectance video and an angiography video. Each reflectance video frame is a simple average of all reflectance B-scans collected at a given lateral position and time point, whereas each angiography video frame was created using the average absolute difference of the magnitude of the complex OCT signal for all reflectance B-scans from the same set.^41^ The angiography processing (average absolute difference), similar to other OCT speckle variance approaches,^31^^,^^34^^,^^42^^,^^43^ separates the static DC OCT reflectance signal from the higher frequency OCT signal that arises from flowing blood. Note that the resulting measurements represent relative blood flow rather than an absolute measurement of blood velocity. The angiography frames were cropped to a 0.4-mm deep region-of-interest (ROI) within the system’s depth of field. We then used the Otsu method^44^ (implemented with the *multithresh* function in Matlab) to segment blood vessels in each angiography frame into three sizes. A representative angiography frame is shown in Fig. 3(b). Once masks were created for each vessel size, the gray-scale values in the angiography frame for all pixels in each segmented region were summed. This value is a measure of the total blood flow in the segmented region, though not a direct measure of flow velocity without further processing.^35^^,^^43^^–^^47^ Therefore, the flow values are normalized to the prestimulation period to generate the temporal response curves in relative units.

### 2.7 Multimodal Synchronization and Data Acquisition Protocol

The OCT system was configured to generate two additional waveforms to synchronize the OCT B-scan acquisition with the median nerve stimulation and the DCS acquisition. The total acquisition time for a single stimulation was 30 s. The first waveform drove the current stimulator with 12 TTL pulses at 0.2 ms duration and 3 Hz (4 s total stimulation duration), starting after a delay of 5 s. The second waveform triggered the DCS system acquisition synchronous to the first (stimulus) waveform. The OCT acquisition was set up to acquire B-scans with 250 A-scans (not including fly-back) at a rate of 240 B-scans/s (4.17 ms interval). In order to collect angiography images of sufficient contrast, 32 B-scans at each lateral location were acquired in 133 ms, where the average absolute difference between B-scans was used to generate the angiography images.^41^ The total acquisition and GPU processing time for each composite B-scan was 460 ms, so the OCT acquisition software was set up to acquire 65 composite B-scans during the 30-s stimulation duration, where the stimulus was on from the ∼11 to 20 B-scans of the acquisition. OCT and DCS acquisition and stimulus waveforms were triggered synchronously.

Each animal was first prepared with anesthesia, coarsely aligned on the stage beneath the objective, and stimulus electrodes were inserted. The focus was set ∼200  μm beneath the window and the window precisely aligned to the OCT beam using the real-time display. Any angle in the window was also adjusted using the two-axis goniometer stage to which the head-bar was affixed. Once aligned, a wide-field OCT scan was acquired to guide probe placement over the somatosensory ROI and further image/data collection. The wide-field 2×2  mm OCT scan (4  μm/pixel) covered the entire cortical region beneath the window and used 10 B-scans at each location to generate the angiography image. The DCS probe was not used and stimulation was not activated during this scan, which took ∼2.5  min to acquire. After the wide-field scan was acquired, the DCS probe was placed over the somatosensory region using the 2×2  mm OCT scan and the surgical photos as a guide. The OCT beam was then aligned through the center channel.

### 2.8 OCT-A En Face Video Survey of Evoked Blood Flow

**Fig. 4:**
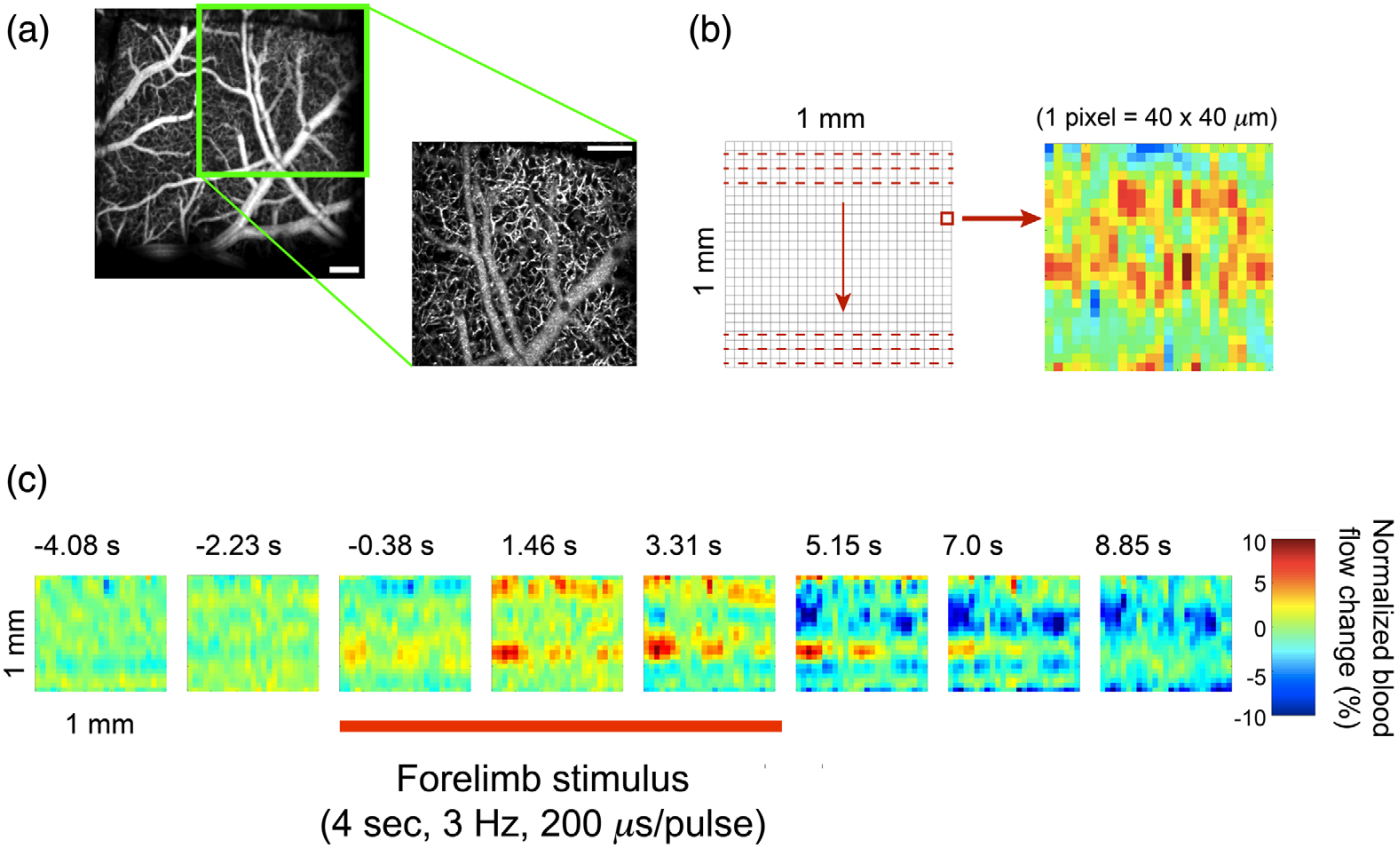
OCT-A *en face* temporal survey of cerebral blood flow patterns following somatosensory stimulation. Scalebar=200  μm. (a) The size of the survey field-of-view relative to the full-field *en face* scan. (b) The general scanning strategy and a representative individual movie frame. (c) Sample extracted OCT-A frames of blood flow patterns during stimulation. The OCT-A survey videos, an example of which is Video 1, have a temporal resolution of 0.46  s/frame. The red bar below the frames indicates the period during which the forelimb was stimulated. Color bar represents changes in blood flow relative to the average of 4 s prior to the stimulus (Video 1, mp4, 645 KB [URL: https://doi.org/10.1117/1.NPh.8.2.025006.1]).

To select the optimal target location for simultaneous acquisition of OCT B-scans and DCS signals during sensory-evoked stimulation, we created an *en face* OCT video survey of blood flow activation during somatosensory stimulation over an ROI spanning 1×1  mm (4  μm/pixel), limited by vignetting of the probe aperture. This region was probed densely in one lateral direction (250 A-scans/B-scan over 1 mm), coarsely in the perpendicular lateral direction (10 B-scan locations over 1 mm), and averaged temporally where 10 somatosensory stimulus presentations were collected at each B-scan location. The *en face* OCT video survey took 50 min to acquire. Data were then processed offline using custom Matlab code (Mathworks Inc., Natick, Massachusetts) to generate an *en face* response survey video as follows. First, at each lateral location, the B-scans collected for the 10 stimuli were averaged. Second, the B-scan were reduced in the dense lateral and axial dimensions by accumulating all pixel values in a 10 (width)×100 (depth) pixel region. This reduced each B-scan region from 250 A-scans (100  pixels/A-scan) to a 25-pixel vector for each location. Third, each of the 65 vectors over time was normalized to the average of the first nine vectors (representing the first 4 s prior to the stimulus onset 5 s after the acquisition start). Finally, the 10 locations in the coarse lateral dimension were expanded by interpolation to 25. This resulted in the final *en face* OCT video where each frame is 25×25  pixels with 65 frames over the 30-s image collection duration. This procedure is depicted in more detail in Fig. S1 in the Supplementary Materials, and an example of the response video is depicted in Fig. 4 and shown in Video 1. It should be noted that the *en face* surveys were used exclusively for selecting the primary recording location, and the actual quantification of vessel diameter-dependent signal was based on the segmented B-scans.

### 2.9 Simultaneous OCT-A and DCS of Evoked Blood Flow

After the response video was examined, the best single lateral location was selected as the ROI, and the animal stages were moved to collect the remainder of the scans during the experimental session. Nine additional OCT B-scan sets spanning 1 mm in one axis (250 A-scans/B-scan) were collected with simultaneous DSC signal acquisition: three trials each at three different evoked stimulus currents (1, 3, and 4 mA, collected in random order), where each trial included 10 stimulus presentations and took 5 min to acquire. The resultant B-scans were averaged over the 10 stimuli, segmented according to vessel diameter with a binary mask using Otsu’s thresholding method over the B-scan focal region (1  mm width×0.4  mm depth), and each timepoint normalized to the average of the corresponding baseline B-scan values (first 4 s) to produce a temporal response curve for each trial. The entire experimental procedure took about 3 to 4 h to complete, including time for setup, alignment, and reconfiguration between scans.

### 2.10 Data Analysis

Experimental data were included in analysis if the animal survived and maintained steady physiological state during the full course of the recording session. This criterion excluded one animal, and an additional animal was excluded from our analysis because of an error during data acquisition. In all, 11 out of 13 animals were included in our data analysis. Significance was tested using two-tailed Student’s t-tests. Normality of the data distribution for any group average was tested using a one-sample Kolmogorov–Smirnov test prior to significance analysis.

## 3 Results

### 3.1 Locating the Centroid of the Hemodynamic Response Using OCT-A

While the mouse somatosensory cortex has a relatively stereotypical representation of the forelimbs,^46^ the exact centroid location of the functional hemodynamic response varies among animals. Given that the OCT-A-measured hemodynamic response waveform can differ significantly depending on the relative distance to the response centroid,^47^ it was important to ensure that our relative recording location was as consistent as possible among all experiments. This presented a challenge given that the peak of the functional response was rapid, on the order of 6 s following stimulus, and obtaining a full *en face* image such as the one shown in Fig. 2(b) required a scan time of 2.5 min (for a single stimulus and with reduced OCT-A averaging). As a compromise, we subdivided the field of view into a grid of subregions, in each of which we performed more dense B-scans, as described in Sec. 2. Because this sparser, more distributed sampling could be achieved relatively rapidly (∼50  min), we were able to effectively create a topological survey of activity (Fig. 4). We used this approach to identify the regions of maximal response and thus select the primary location to acquire higher pixel density B-scans.

### 3.2 Sensory-Evoked Blood Flow in the Cerebral Cortex Varies as a Function of Vessel Diameter

As shown in Fig. 3, OCT-A imaging resolved cross-sections of blood vessels of different diameters. Using Otsu’s thresholding method,^44^ we created binary masks that were then used to dissect flow measurements from different vessel populations, including diameters of 10 to 20  μm and >20  μm. Because the axial and lateral resolutions of the OCT-A device were ∼9  μm, smaller features were not well resolved using thresholding. Consequently, the regions of the B-scans that yielded non-zero flow but did not overlap with other vessel populations were attributed to vessels of diameter <10  μm.

**Fig. 5:**
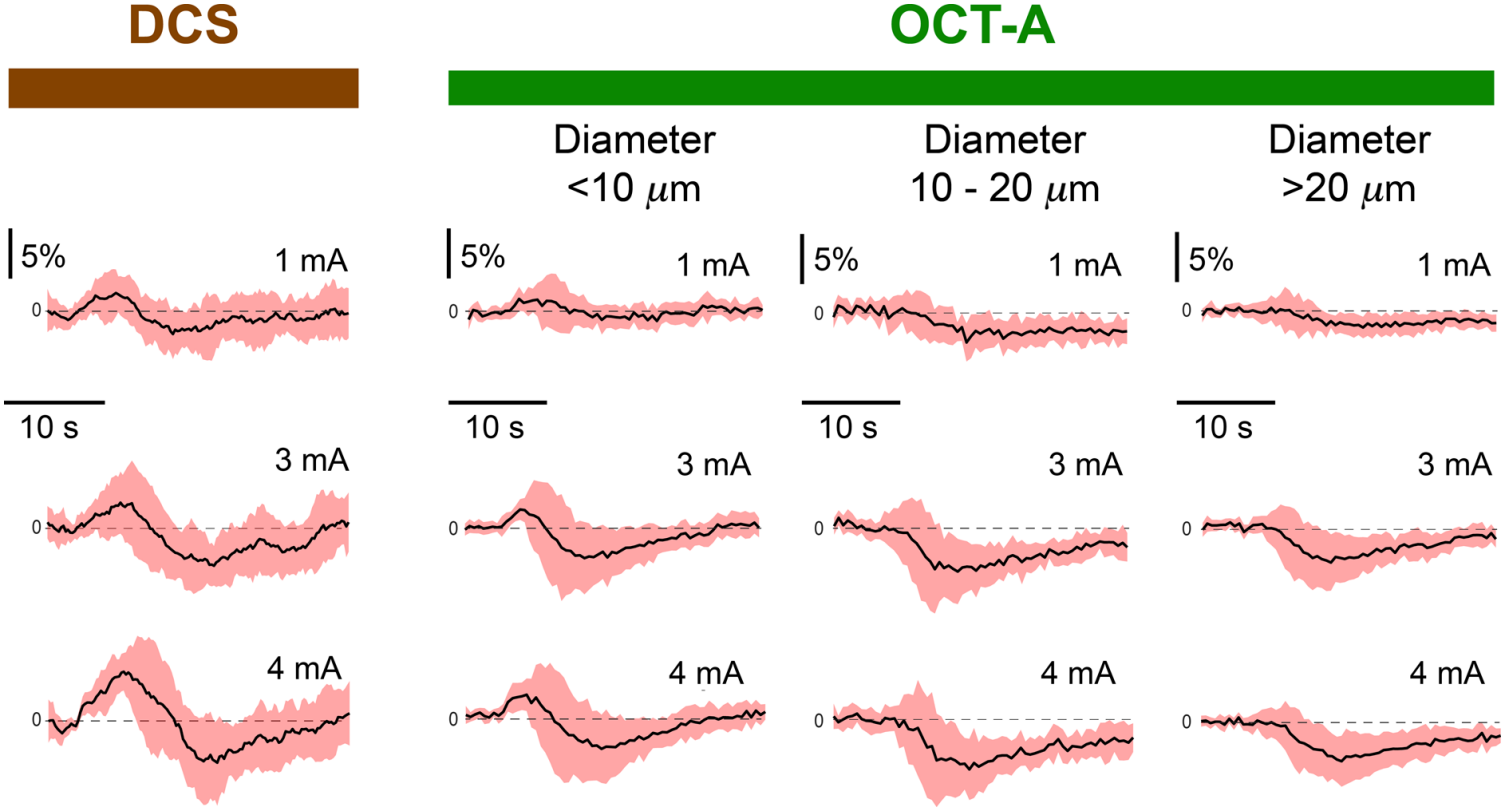
Image segmentation-based dissection of OCT-A signals reveals that hemodynamic response features depend on vessel diameter. Traces on the left-most column depict DCS measurements of somatosensory-evoked hemodynamic responses from the grand average of all experiments. Each trace represents the average of 11 animals, and the three traces aligned vertically depict the responses to increasing stimulation current intensities. The three columns of traces on the right depict spatially segmented components of the OCT-A response. The traces at each stimulus current for both DCS and OCT-A were recorded simultaneously.

**Fig. 6:**
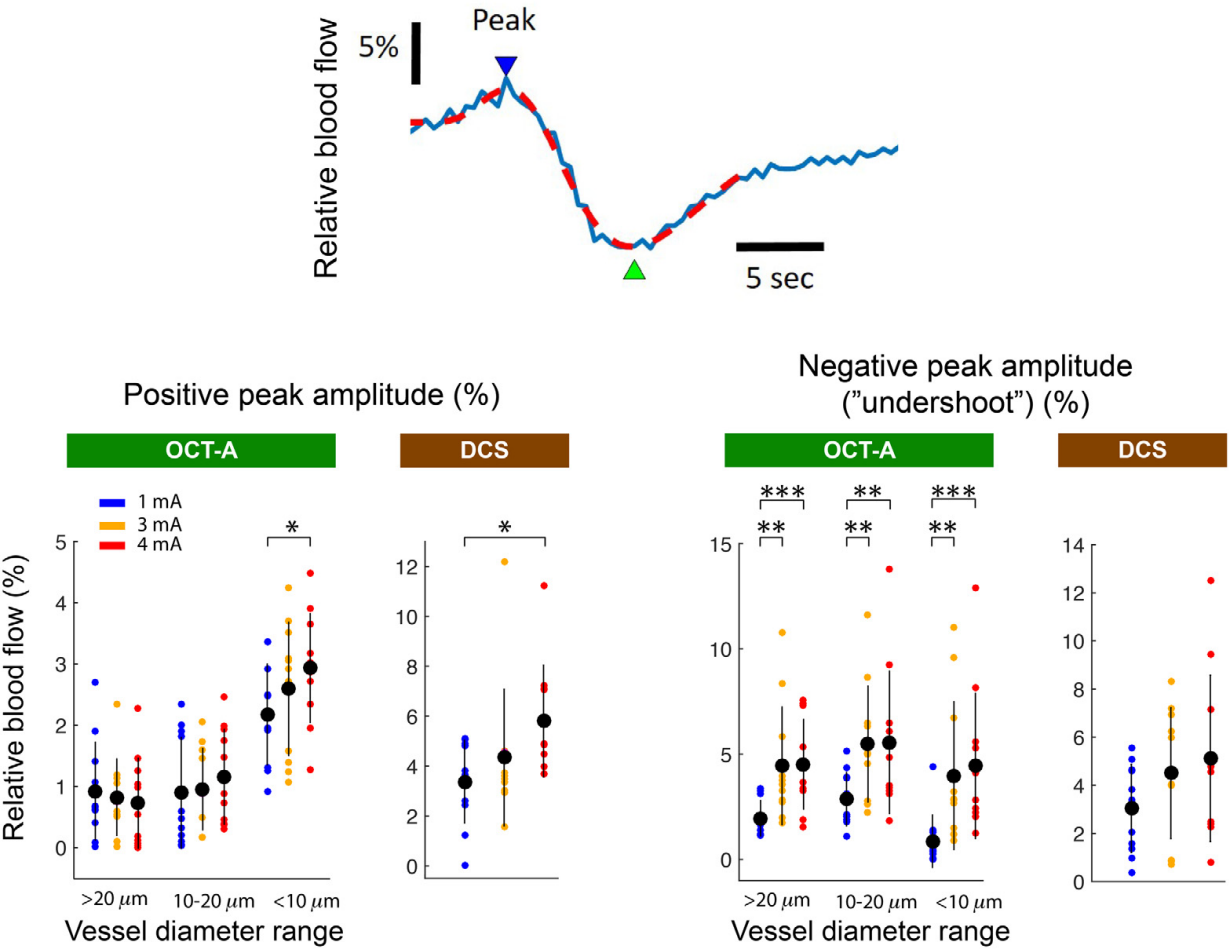
Side by side comparison of simultaneously measured DCS and OCT-A hemodynamic responses, averaged across all animals (n=11). The top waveform depiction illustrates how waveforms were quantified; the red dashed line is a dual gamma function fit (see Sec. 2) to the evoked DCS blood flow response (blue trace) in one experiment, which was selected for illustration because of its prominent biphasic features. The blue and green arrows show the peak locations at which amplitudes were assessed. Note that the exact time points varied from experiment to experiment depending on the fit. Analyzed using image segmentation, putative vessels of <10  μm diameter in OCT-A imaging results featured the most prominent positive initial peaks, which occurred 7.3±0.3s after the stimulus (mean±standard deviation). Putative vessels of larger diameter were relatively unresponsive during the initial positive phase and did not demonstrate significant stimulus intensity dependence. The amplitude of the subsequent undershoot, however, was greater in these putative vessels with larger diameters. In DCS signals, the initial positive peaks demonstrated a greater stimulus intensity dependence between 1 and 4 mA compared with the responses during the undershoot period. Error bars represent mean±standard deviation. The brackets and asterisks above the data points depict the statistical significance of the difference in the grand averaged response compared with the response to 1 mA stimulation. Asterisks indicate the P-value from a two-tailed Student’s t-test as follows: * indicates P<0.05, ** indicates P<0.01, *** indicates P<0.001.

Integrating the sensory-evoked blood flow selectively in the three vessel diameter ranges within the OCT-A recordings yielded hemodynamic responses with qualitatively different waveform attributes (Fig. 5). Averaged among all animals (n=11), the most prominent features for both DCS and OCT-A measurements were positive and negative peaks at ∼8 and ∼15  s, respectively. The relative flow amplitude of the positive peak, resolved by OCT-A at a stimulation current of 1 mA, was 2.19±0.8% (mean±SD) in the areas putatively representing vessels of diameter <10  μm (Fig. 6). This was more than double the response summed among regions putatively representing the largest vessel diameters, 0.92±0.8%. Meanwhile, for the negative peak, the amplitude magnitude in the same large vessel population 1.96±0.86% was ∼2.3 times greater than the flow in the putatively <10  μm diameter vessels (0.86±1.26%). Overall, in our experiments, the maximum positive peak amplitudes in OCT-A were found for the <10  μm diameter group at a stimulus current of 4 mA, whereas the maximum amplitude for the undershoot was found in the 10- to 20-μm diameter vessel group at a stimulus current of 3 mA.

**Fig. 7:**
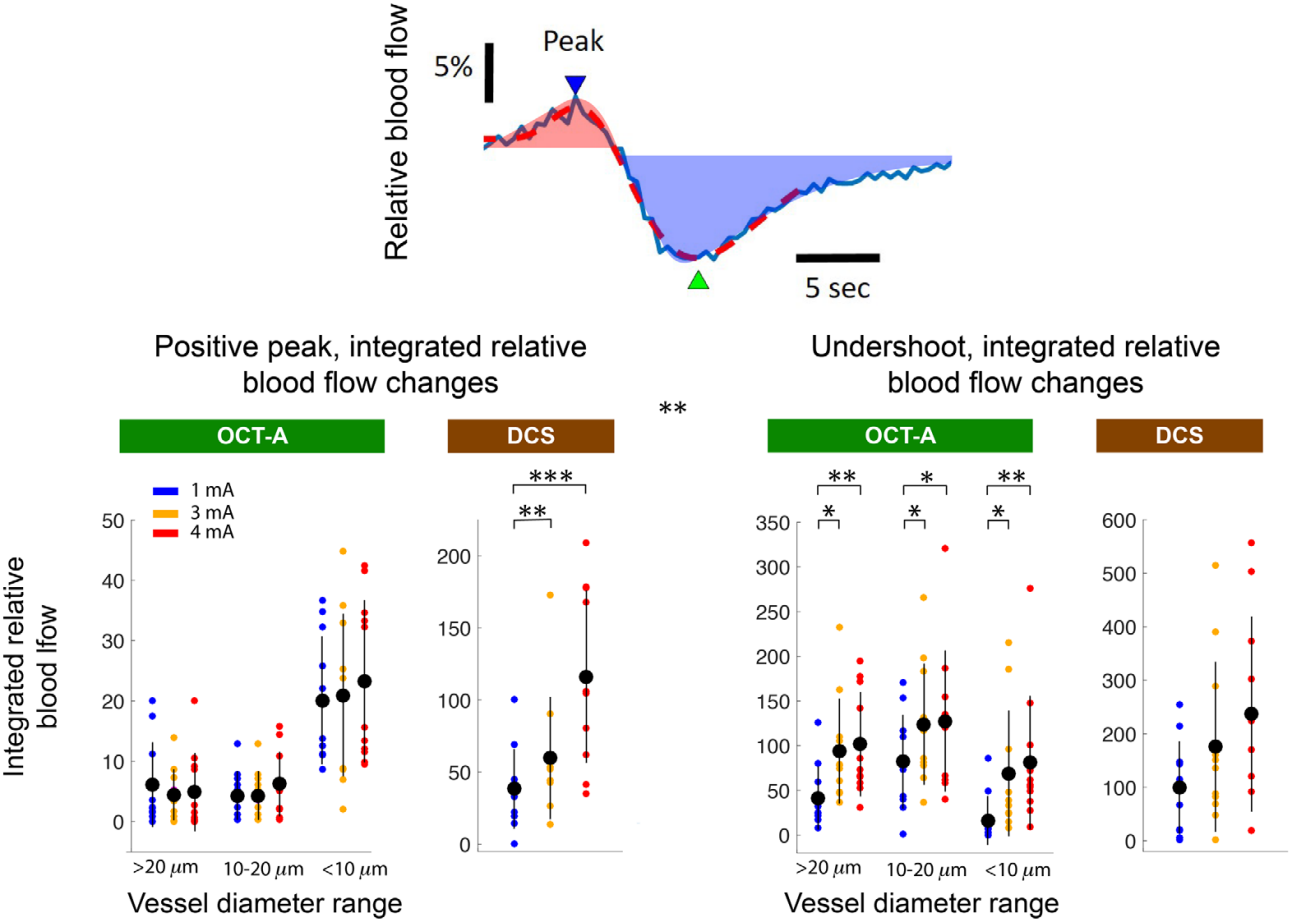
Integrated waveform features accentuate the diameter-dependence of response amplitude during positive and negative peaks post stimulus. As in Fig. 5, the top DCS waveform depiction illustrates how waveforms were quantified. The dashed red trace superimposed on the blue trace is a double gamma function fit. Superimposed on the curve are red and blue shaded regions that correspond to the positive and negative peaks, respectively. These regions are defined by the points at which the blood flow waveform crosses above/below the baseline value. The exact time points that defined these positive and negative phases varied from experiment to experiment depending on the fit. Bar charts represent the average over all animals (n=11). Error bars represent mean ± standard deviation. The use of brackets and asterisks is as in Fig. 5.

Compared with the blood flow waveforms measured in small vessels with OCT-A, the blood flow waveforms obtained through DCS had a more pronounced undershoot. For example, at 1-mA stimulus current in OCT-A measurements, the positive peak amplitude of the putatively <10  μm diameter vessels was ∼2.5 times greater than the vessel population’s average undershoot amplitude. At the same stimulus current, the positive peak magnitude of the DCS signal was only ∼10% greater than the measurement undershoot (3.37±1.67% and −3.04±1.83%, respectively). As an additional contrast, while the magnitudes of the DCS signal were within an order of magnitude of the OCT-A counterparts, the DCS peaks were broader. This can be visualized in terms of the integrated signal, as seen in Fig. 7. The difference was most prominent for the undershoot, where the integrated DCS values were larger than their OCT-A counterparts at any stimulus current and vessel diameter population.

### 3.3 Correlation Between Evoked Blood Flow Waveforms in DCS versus OCT-A-Derived Vessel Subpopulations

**Fig. 8:**
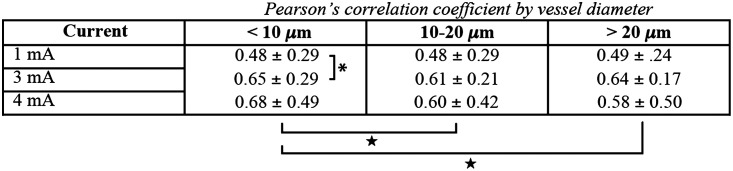
Correlation values, averaged over all animals, of DCS versus OCT-A flow waveforms integrated among putative vessel subpopulations of differing diameter. The values represent Pearson’s correlation coefficient comparing full, 30-s waveforms. Correlation coefficient values were moderate for all vessel populations, and highest among the small vessels. The putatively <10  μm diameter vessels demonstrated a significant increase in correlation coefficient when forelimb stimulus intensity was increased from 1 to 3 mA. Values are mean±standard deviation, and the asterisk* indicates that P<0.05 when using a two-tailed Student’s t-test to assess statistical differences between the correlation coefficient mean±standard deviation of the 3 mA response versus that of the 1 mA response. ★ indicates that the correlation coefficient (±standard deviation) differs significantly from that of the <10  μm diameter vessels at 4 mA stimulus current, also assessed using a criterion of P<0.05 obtained through a two-tailed Student’s t-test.

As shown in Fig. 5, the flow waveform varied considerably between different vessel populations. As one strategy for assessing underlying vascular architectural substrates for the DCS signal, we calculated the Pearson’s correlation coefficient of DCS waveforms with the concurrently recorded OCT-A signals. Figure 8 depicts correlation values, averaged over all animals, of DCS versus OCT-A flow waveforms integrated among putative vessel subpopulations of differing diameter. Overall, the mean correlation coefficients for all three vessel diameter ranges increased when the stimulus current was increased from 1 mA. The highest correlation value, 0.68±0.49, was observed in the putatively <10  μm diameter population at a stimulus current of 4 mA. Within this small diameter vessel population, the correlation value measured at a stimulus current of 3 mA represented a significant increase in correlation coefficient relative to the waveform elicited by 1 mA stimulus current (P=0.04 using a two-tailed Student’s t-test). At 4-mA stimulus current, the correlation values of the 10 to 20  μm and >20  μm groups were each statistically different from that of the <10  μm diameter group (P<0.05 using a two-tailed Student’s t-test).

## 4 Discussion

The sensitivity of DCS to cerebral blood flow in microvasculature makes it an attractive neuromonitoring modality. Similar to the challenges that have historically limited the clinical implementation of quantitative EEG (qEEG),^48^ a solid understanding of how to interpret the recorded signals is key. For example, DCS has been implemented clinically for monitoring the microvascular response to ischemic stroke in relatively large vessels.^5^^,^^21^ What might be the limits on DCS sensitivity to detecting ischemia in smaller vessels? From a translational standpoint, understanding practical limitations could also help medical device regulators better assess the technology. Our work adds a direct, simultaneous exploration of the activity in fine vessels, putatively the basis for the DCS signal.^10^^,^^16^^,^^25^^,^^49^

At an empirical level, the optical signals we observed are similar to prior measurements that have used DCS to observe sensory-evoked blood flow responses. The magnitudes of the positive peaks we observed are comparable to the ∼4% changes observed during somatosensory stimulation in human infants.^18^ Compared with our own previous work in mice, however, the amplitudes we measured in this study were roughly 2× smaller.^36^ This deviation is likely attributed to a difference in surgical procedures; whereas our previous report involved only minor acute surgery featuring a relatively small burr hole, a chronically implanted window was required to obtain OCT-A access over a larger area. Although we were careful to minimize trauma and animals recovered well with no apparent behavioral side effects, residual bleeding, or bruising, it is possible that there was some level of trauma and compromise of the microvasculature due to the craniotomy itself.^50^ Regarding the biphasic nature of the evoked responses and the presence of a prominent undershoot, our findings match those empirically observed in the other DCS studies as well as evoked responses measured by other modalities, such as laser speckle,^51^^,^^52^ laser Doppler,^53^^,^^54^ and arterial spin labeling.^55^

As for our OCT-A measurements of sensory-evoked blood flow, the results are qualitatively comparable with previous work. Both Srinivasan et al.^49^ and Shin et al.^56^ observed biphasic flow responses in small vessels that featured positive flow changes of ∼4% to 5%. The positive peak flow amplitudes we observed in small vessels were ∼30% smaller, however, and flow in vessels with diameter >10  μm was far lower. We believe several factors may have contributed to this. One explanation is an oversampling of venous flow, given that we did not distinguish arterial and venous flow in our OCT-A measurements. In terms of the underlying physiology of flow changes, small arterioles are at the frontline of neurovascular coupling. Neuronal activity activates smooth muscle cells lining nearby arterioles through an intricate choreography involving multiple cell types and signaling mechanisms, collectively termed the neurovascular unit.^57^ The resulting dilatory or contractile mechanical changes backpropagate up through the vascular tree to larger arterioles and arteries, likely through signaling pathways that travel through vascular endothelial cells. Dilation in venules and veins, in contrast, is muted and delayed.^53^^,^^54^ The actual diameter range of small penetrating arterioles and venules overlaps,^58^ so if vessel type is not discerned, it is logical that even the small vessel response, which was binned by diameter, would appear blunted.

Smaller positive peak amplitudes in the OCT-A recordings could also have been influenced by our method of identifying the centroid of the cortical response. Our *en face* functional survey method (Fig. S1 in the Supplementary Materials) involved performing a series of B-scans centered at 10 lateral locations. Each A-scan of a B-scan was projected to a single pixel whose amplitude was obtained by integrating the signal through vessels along the entire depth of that location’s A-scan. The volume was further resampled in both lateral dimensions. Our *en face* surveys were therefore coarse samplings of the volume and could have produced an inaccurate prediction of the true response centroid location. Again, the pronounced undershoot we observed in the OCT-A signal, and its correlation with the DCS signal, supports the hypothesis that our measurement location deviated somewhat from the response centroid given that undershoot increases with distance from the response centroid.^45^

For larger vessels, which were predominantly superficial, it is also possible that the blunted positive flow response was due to alterations in tissue optical properties not directly related to flow. Tang et al. (2020),^59^ for example, observed a transient reduction in the superficial tissue refractive index upon whisker stimulus in mice that peaked roughly 10 s after the stimulus onset. This effect, posited as related to changes in the interstitial fluid-filled spaces in the regions surrounding vessels (the Virchow–Robin space), coincides fairly well with our observed undershoot phase. It is possible, albeit improbable given that our OCT-A flow measurements are insensitive to slow “offset” effects, that these optical changes in some way influenced the OCT-A readout of flow and amplified the apparent flow undershoot.

Modeling work has yielded significant insight into the biophysical underpinnings of DCS measurements. In particular, modeling has addressed some counterintuitive trends. Myriad imaging studies have observed that red blood cells exhibit rigid body collisions amid flow within a network of vessels.^60^ However, models that yield the best fit to DCS measurements assume that red blood cell displacement follows Brownian diffusion, rather than random ballistic trajectories.^17^ Carp et al.^61^ suggested that the phenomenological diffusive component of red blood cell motion could instead be due to photons that scatter multiple times within single blood vessels; this implies that the DCS originates from blood vessels of diameter large enough to sustain multiple photon scattering events, typically >12  μm at the wavelengths used here and in most other DCS instrumentation. A more recent follow-up study, however, found that the diffusive quality of the measured photon dynamics can be largely attributed to shear-induced relative motions of red blood cells due to hydrodynamic interactions.^62^ While the study was able to characterize how vessel diameter affects blood flow calculations, it was not possible to implicate a specific diameter range of vessels because the DCS signal has a complex, nonlinear dependence on the overall range of vessel diameters within the tissue, their relative spatial densities, as well as the red blood cell speed within each vessel type. Although our OCT-A measurements did not afford a detailed investigation of vessel diameter distributions given that only one B-scan location was selected for recording, our correlation findings suggest that the signal is biased toward vessels whose diameter is closer to the original lower bound (>12  μm) posited by Carp et al.^61^ It remains possible, however, that this trend is specific to small source–detector separations, such as the 5 mm used in this study. The fact that the DCS waveforms were most significantly correlated with the OCT-A waveforms in response to 3-mA stimulus current likely reflects the fact that increasing stimulus intensity changed peak-to-undershoot symmetry. When comparing DCS and OCT-A results, the peak/undershoot ratios for the <10  μm diameter vessel population matched best at 3-mA stimulus current.

Finally, the difference in spatial resolution of the two optical modalities complicates interpretation. Whereas calculating the spatial resolution of imaging devices such as our OCT-A system is straightforward, spatial resolution is far more challenging to quantify for diffuse optical techniques. For diffuse methods, detectible “signal” can originate from any location in the brain, skull, or scalp. However, for a single source–detector pair, one way to quantify spatial resolution is in terms of depth-dependent sensitivity. For example, Strangman et al.^63^ found that for source–detector separations 10 mm and below, fNIRS sensitivity is significantly biased toward signals originating from within a depth of ∼5 to 10 mm. In comparison, the spatial resolution of our OCT-A device was upward of three orders of magnitude finer. While the physical principles of DCS, presumably echoed in our results, bias the signal toward flow in smaller vessels, the DCS signal most likely reflects hemodynamics from the entirety of the forelimb’s representation on the cortex, which spans an area with a diameter of ∼2  mm.^64^ Based on the results of Srinivasan and Radhakrishnan^49^ as well as findings from other modalities,^25^^,^^26^^,^^65^^,^^66^ the DCS signal would have integrated flow signals from vessels both near the cortical sensory response’s centroid as well as from vessels in the surrounding cortical regions, which feature prominent undershoots. Based on these spatial considerations, the undershoot observed in our DCS signals likely reflects this summation. Given our OCT-A system’s spatial resolution as well as limitations on discerning vessel type, a definitive breakdown of arteriolar, venous, and capillary contributions to the DCS-recorded hemodynamic response is still pending.

## 5 Conclusion

Overall, this study presents a multiscale investigation of blood flow in the fine vasculature of the cerebral cortex together with a diffuse technique that is purportedly biased to signal in those very vessels. The initial positive hyperemic peak that we observed in the DCS response appears to most significantly reflect flow in vessels of diameter <10  μm, which includes small arterioles, venules, and capillaries. On an application level, our results support the assertion that DCS fills a specific niche in bedside neuromonitoring given that flow disruption in small vessels can precede more severe, larger scale ischemia. Because of the significant difference in the integration volume of both modalities, future experiments involving larger animals, the use of multiple DCS source–detector pairs, and flow direction sensitive, higher spatial resolution OCT-A measurements acquired over a broader area can help overcome some of these interpretation challenges.
