# Supplementary material for: Dissecting the microvascular contributions to diffuse correlation spectroscopy measurements of cerebral hemodynamics using optical coherence tomography angiography
Source: Neurophotonics. 2021 Apr 25;8(2):025006. doi: 10.1117/1.NPh.8.2.025006 (PMC8071783; doi:10.1117/1.NPh.8.2.025006)
Supplement: Supplementary file 2 [file NPh_008_025006_SD002.pdf]

Supplementary Materials

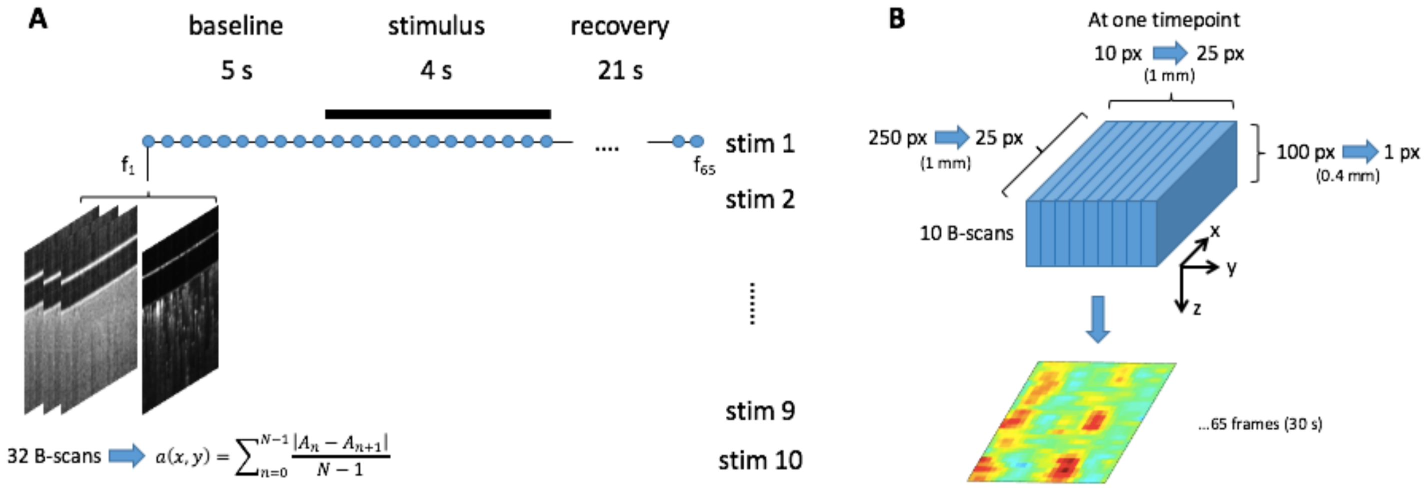

Supplemental Figure 1:

Schematic depicting generation of OCT-A response signal and survey video. A For each stimulus presentation, 65 frames were collected over 30 s, where each frame is the angiography map generated from 32 B-scans collected from the same lateral location. The absolute average difference algorithm was used to generate the angiography image as shown in the equation. 10 stimuli were presented and averaged, and the average baseline was subtracted from each timepoint to create the response signal at one location. B For the survey video, the timing protocol shown in A was repeated at 10 locations. A single frame of the survey video (one timepoint) was created by decimating the set in the x-axis (B-scan direction), expanding the set in the y-axis from 10 to 25 pixels with spline interpolation, and projecting the z-axis to one plane (equivalent to an average intensity projection). The complete survey video (see Fig. 4 and Supplemental Video) is created at all 65 timepoints where the interval is 0.46 s.
